# The SIRT2-AMPK axis regulates autophagy induced by acute liver failure

**DOI:** 10.1038/s41598-024-67102-w

**Published:** 2024-07-15

**Authors:** Qingqi Zhang, Jin Guo, Chunxia Shi, Danmei Zhang, Yukun Wang, Luwen Wang, Zuojiong Gong

**Affiliations:** Department of Infectious Diseases, Renmin Hospital of Wuhan University, Wuhan, 430060 China

**Keywords:** Hepatology, Inflammation

## Abstract

This study explores the role of SIRT2 in regulating autophagy and its interaction with AMPK in the context of acute liver failure (ALF). This study investigated the effects of SIRT2 and AMPK on autophagy in ALF mice and TAA-induced AML12 cells. The results revealed that the liver tissue in ALF model group had a lot of inflammatory cell infiltration and hepatocytes necrosis, which were reduced by SIRT2 inhibitor AGK2. In comparison to normal group, the level of SIRT2, P62, MDA, TOS in TAA group were significantly increased, which were decreased in AGK2 treatment. Compared with normal group, the expression of P-PRKAA1, Becilin1 and LC3B-II was decreased in TAA group. However, AGK2 enhanced the expression of P-PRKAA1, Becilin1 and LC3B-II in model group. Overexpression of SIRT2 in AML12 cell resulted in decreased P-PRKAA1, Becilin1 and LC3B-II level, enhanced the level of SIRT2, P62, MDA, TOS. Overexpression of PRKAA1 in AML12 cell resulted in decreased SIRT2, TOS and MDA level and triggered more autophagy. In conclusion, the data suggested the link between AMPK and SIRT2, and reveals the important role of AMPK and SIRT2 in autophagy on acute liver failure.

## Introduction

Acute liver failure (ALF) is a critical clinical condition marked by rapid hepatocyte function deterioration in individuals without prior liver disease. It can result from various factors, including prolonged ischemia, toxic exposure, drug reactions (both idiosyncratic and dose-dependent), tumors, metabolic disorders, as well as infectious and immune-mediated processes^1^. Currently, effective treatments for ALF are limited.. Autophagy, a lysosome-mediated degradation process, is essential for cellular and metabolic balance in the liver^2^. It is intricately linked to various liver diseases, such as drug-induced liver injury (DILI)^3^, viral hepatitis^4^, and non-alcoholic fatty liver disease^5^, all of which are associated with autophagy impairment.

Autophagy involves sophisticated molecular pathways that transport intracellular components to lysosomes for breakdown and reuse, crucial for maintaining cellular and organismal homeostasis. Molecular pathways central to the maintenance of cellular and organismal homeostasis by autophagy^6^. In mammalian cells, autophagy initiation requires the activation of UNC-52-like kinase 1 (ULK1) or ULK2 at the phagophore assembly site (PAS). AMP-activated protein kinase (AMPK), a sensor of intracellular ATP levels, comprises a catalytic subunit (PRKAA/AMPKα) and two regulatory subunits (PRKAB/AMPKβ and PRKAG/AMPKγ), the α-subunit contains a catalytic kinase domain and exists as either the α1 or α2 isoform^7^. When energy is low, AMPK activates and phosphorylates ULK1 to promote autophagy^8^. Beclin1, part of the class III phosphoinositide 3-kinase (PtdIns3K) complexes, also facilitates autophagy^9^. Notably, acetaminophen (APAP) overdose induces autophagy, and its pharmacological activation can prevent APAP-induced liver injury^10^. Lipocalin prevents APAP-induced hepatotoxicity by activating AMPK and ULK1-mediated autophagy^11^. Numerous studies have demonstrated a close link between PRKAA1 and autophagy^12,13^. In present study, we would like to explore the expression of PRKAA1 in ALF, as well as to study the link between PRKAA1 and SIRT2.

Sirtuin 2 (SIRT2), a NAD + -dependent deacetylase, predominantly resides in the cytoplasm but also exists in mitochondria and the nucleus^14^. Recent studies highlight its significant role in various liver diseases, including hepatocellular carcinoma^15^, alcoholic liver disease^16^, and non-alcoholic fatty liver disease17. Tang et al. demonstrated that SIRT2 binds to liver kinase B1 (LKB1), deacetylating it at lysine 48, thereby enhancing LKB1 phosphorylation and the subsequent LKB1-AMPK signaling activation^18^. Moreover, SIRT2 also impedes cardiac hypertrophy through LKB1 deacetylation and the activation of LKB1-AMPK signaling^19^. SIRT2 may achieve regulation of autophagy by modulating the LKB1-AMPK axis. SIRT2 appears to play different roles in acute and chronic diseases. In the mice model of cisplatin-induced acute kidney injury, SIRT2 knockout mice showed improvements in apoptosis, necrosis, and inflammation^20^. In acute liver failure, the pathways by which SIRT2 regulates autophagy are unknown. Our previous study has verified that SIRT2 inhibitor AGK2 possesses hepatoprotective properties via regulating the MFN2-PERK axis and ferroptosis signaling pathway^21^. Here, we aimed to explore how SIRT2 influences autophagy in AML12 cells and mice with TAA-induced ALF, focusing particularly on its interaction with AMPK.

## Materials and methods

### Reagents and antibodies

We sourced AGK2 from Selleckchem (USA) and thioacetamide (TAA) from Sigma (St. Louis, USA). Plasmids including pLV3-CMV-PRKAA1-Puro, pLV3-CMV-SIRT2-Puro, pLV3-U6-PRKAA1-shRNA-Puro, and pLV3-U6-SIRT21-shRNA-Puro were acquired from Miaoling Biology (Wuhan, China). The pCMV-mCherry-GFP-LC3B plasmid was obtained from Beyotime Institute of Biotechnology (Wuhan, China). Hieff Trans® Liposomal Transfection Reagent was purchased from Yeasen Biotechnology (Shanghai, China). Antibodies targeting SIRT2, PRKAA1/AMPKα1, P-AMPKα1, LC3B, P62, Beclin1, and GAPDH were procured from Abcam (Cambridge, MA, USA). Dulbecco's Modified Eagle Medium (DMEM) and Fetal Bovine Serum (FBS) were from Gibco (NY, USA). The Malondialdehyde (MDA) assay kit came from Naijing Jiancheng Bioengineering Institute (Naijing, China), and Total Oxidant Status (TOS) kits were sourced from Elabscience (Wuhan, China).

### Animals

Male C57BL/6 wild-type mice (n = 30) specific pathogen-free (SPF) (20—25 g) were purchased from Experimental Animal Center of Wuhan University. Protocols of this study were approved and consented on by the Ethics Committee of Renmin Hospital of Wuhan University. The mice were fed in the animal experiment center in Renmin Hospital of Wuhan University. They were acclimated for 1 week before modeling. 30 mice were divided randomly into 3 group: normal (n = 10), model (n = 10), AGK2 group (n = 10). The sample size of mice was obtained by referring to other acute liver failure literatures^22–25^. The AGK2 (1 μmol/mouse) was injected into abdominal cavities in mice in AGK2 group. The same volume of normal saline was given in normal group mice. After 2 h, mice in the model group and AGK2 group were induced by intraperitoneal injection of TAA (600 mg/kg). The doses of TAA were administrated according to previous studies^26,27^. All experimental mice were sacrificed under anesthesia after 24 h when TAA administration. All animals received care in accordance with the recommendations of the National Institutes of Health Guide for Care and Use of Laboratory Animals and adhered to the ARRIVE guidelines.

### Cell culture and transfection

The mouse liver cell line AML12 obtained from China Center for Type Culture Collection (CCTCC) were grown in Dulbecco’s Modified Eagle’s Medium (DMEM) medium containing 10% fetal bovine serum (FBS), incubated at 37℃ in a 5% CO2 atmosphere. First, Different TAA concentrations (0 mM, 30 mM, 60 mM, 90 mM, 120 mM, 150 mM) were used to stimulate AML12 cells to detect the cell death rate and select the appropriate concentration of TAA for ALF modeling in vitro experiment. Then cells were categorized into normal, TAA, and TAA + AGK2 groups. Excluding the normal group, TAA (90 mM) was added to the media of each group for 24 h. In the TAA + AGK2 group, AGK2 (1 μM) was introduced to the medium 2 h prior to TAA (90 mM) addition.

### The cell death rate of AML12 cells was measured

The cell death rate of AML12 cells was determinated by a kit (BD, USA). The dosage of TAA (0 mM, 30 mM, 60 mM, 90 mM, 120 mM, 150 mM) was given to each well of a 6-well plate. Cells were collected after 24 h when TAA administration. Then the cells were washed twice, added with 5 μL PE Annexin V and 5 µl 7-AAD, and gently vortexed and incubated. Finally, add 400 µl of 1 × Binding Buffer and the cell death rate was measured by flow cytometry (BD, USA).

### Lentiviral vectors transduction for SIRT2 and PRKAA1 knockdown

Plasmids pLV3-U6-PRKAA1-shRNA-Puro (sh PRKAA1) and pLV3-U6-SIRT21-shRNA-Puro (sh SIRT2), along with an empty vector (LKO), were transfected into 293 T producer cells using Hieff Trans® Liposomal Transfection Reagent, following the manufacturer’s protocol. After 48 h of culture, supernatants were collected and concentrated via ultracentrifugation. The packaged sh PRKAA1, sh SIRT2, and LKO were then transfected into AML12 cells. Transfectants underwent selection with puromycin, and the efficiency of SIRT2 and PRKAA1 knockdown was verified by western blot analysis.

### Lentiviral vector transfection for SIRT2 and PRKAA1 overexpression

We utilized pLV3-CMV-PRKAA1-Puro (OE PRKAA1) and pLV3-CMV-SIRT2-Puro (OE SIRT2), along with an empty vector (CMV) for transfection. These were transfected into 293 T producer cells alongside packaging vectors. The transfection procedure followed is the same as described in Sect. 2.4.

### Histopathological analysis and biochemical examination

For histopathological analysis, a portion of the fresh liver specimens from mice was fixed in 10% neutral-buffered formalin, embedded in paraffin, sectioned, and stained with hematoxylin–eosin (HE). These sections were then examined under a BX 51 light microscope (Olympus, Japan). Another portion of the liver specimens was fixed in 4% glutaraldehyde, dehydrated with ethanol and acetone, embedded in epoxy resin, sectioned, and stained with saturated uranium acetate and lead citrate for ultrastructural observation under a transmission electron microscope (HITACHI HT7700, Japan). Serum alanine aminotransferase (ALT) and aspartate aminotransferase (AST) levels in each group were measured using an automated Aeroset chemistry analyzer (Abbott Co. Ltd., USA).

### Western blotting

Proteins (30 μg) extracted from the cells were separated on 12% SDS-PAGE and transferred onto polyvinylidene fluoride (PVDF) membranes. Considering proteins with similar molecular weight sizes, membranes were then cut according to the sizes of proteins to ensure that all PVDF bands were transferred and tested in the same Wb experiment. After blocking with 20% non-fat milk for 1 h, the membranes were incubated overnight at 4 °C with primary antibodies against P-AMPKα1 (1:1000), P62 (1:10,000), LC3B (1:2000), Beclin1 (1:1000), and GAPDH (1:2000). Subsequently, they were incubated with HRP-conjugated Affinipure Goat Anti-Rabbit IgG(H + L) (1:5000) for 1 h. Protein bands were visualized and quantified using QuickChemi 5200.

### Immunofluorescence assay

Cells subjected to specific treatments were fixed with 4% paraformaldehyde for 30 min and permeabilized with 0.5% Triton X-100 for 15 min. After washing with ice-cold phosphate-buffered saline (PBS) and blocking with 0.5% bovine serum albumin (BSA) in PBS for 30 min, cells were incubated with rabbit polyclonal antibodies against SIRT2, PRKKA1, and P-PRKKA1, followed by treatment with TRITC-conjugated goat anti-rabbit IgG antibody. Nuclei were stained with DAPI. The cells were then visualized using a fluorescence microscope (Olympus, Japan, #BX53).

### pCMV-mCherry-GFP-LC3B assay

pCMV-mCherry-GFP-LC3B plasmid is often transfected into cells to analysis the autophagy level. GFP puncta were used a confocal microscopy (Olympus, Japan, #FV1200).

### Detection of MDA and TOS

The MDA and TOS kits were performed according to the corresponding manufacturer's instructions. The values for the levels of MDA and TOS in the AML12 cells and liver tissue were detected with the multimode plate reader.

### Statistical analysis

Statistical analysis and graph generation were performed using GraphPad Prism 9.5. Data normality was assessed using the Shapiro–Wilk test, and homogeneity of variances was tested using Levene’s test. Multiple data sets were analysed using Analysis of Variance (ANOVA) and post hoc tests. Least Significant Difference was used for variance-aligned data , and the Brown-Forsythe test was used for variance-discrepant data. P < 0.05 was designated as significant.

## Results

### AGK2 ameliorated pathological damage in ALF mice

**Figure 1:**
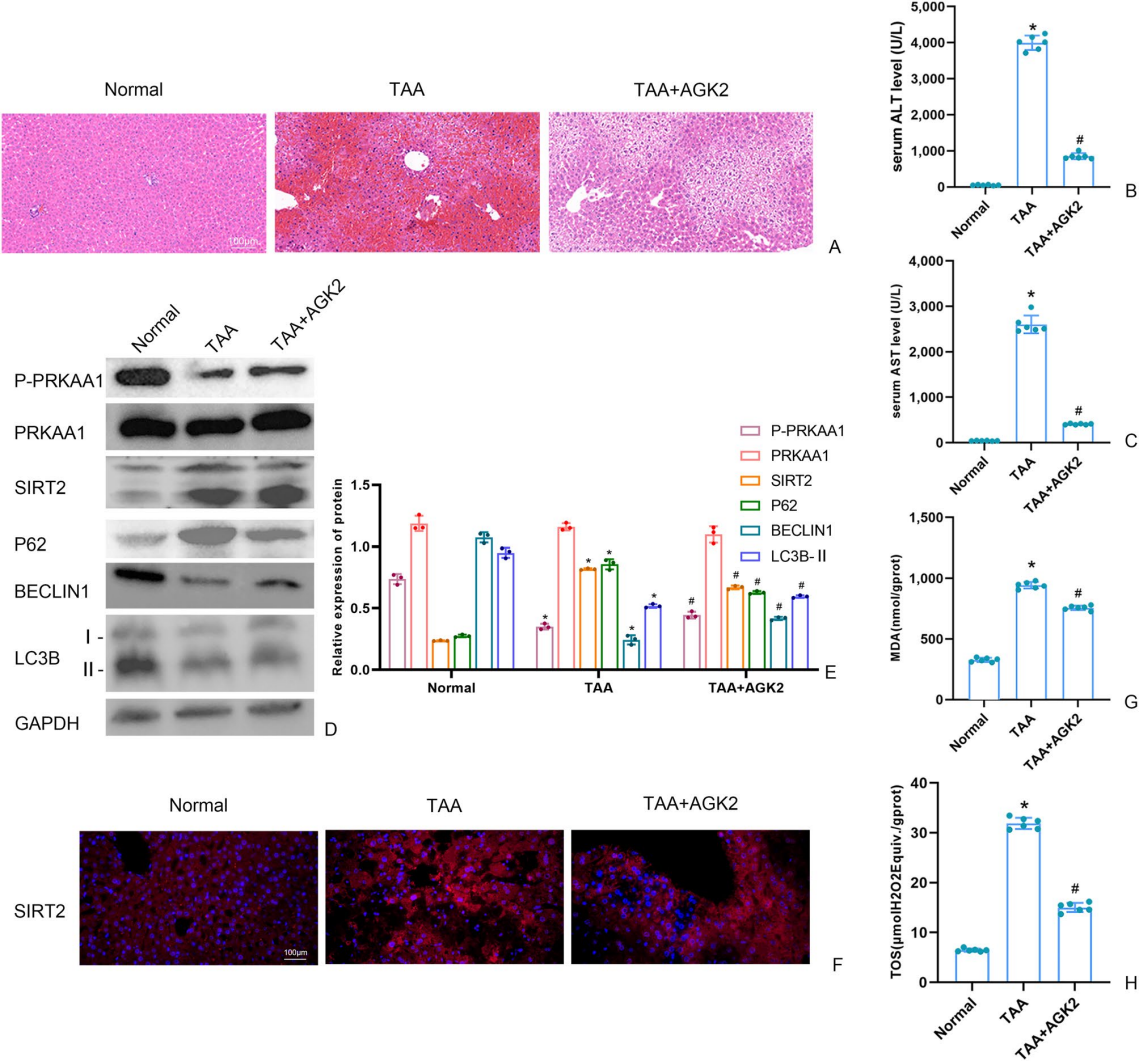
(**A**) The liver tissues were stained with HE (scale bars = 100 μm). (**B**) The fluorescence intensity of liver SIRT2 by immunofluorescence (scale bars = 100 μm). (**C**,** D**) The SIRT2 protein and autophagy protein level in liver were measured by Western blot and quantitatively analyzed. Original blots are presented in Supplementary Figure S1. (**E**,** F**) The serum level of ALT and AST in liver. (**G**,** H**) Malondialdehyde (MDA) and Total Oxidant Status (TOS) levels in AML12 cells detected by kits. Data were expressed as the mean ± SD. P values were determined by 2-tailed Student t test. Compared with normal group, *P < 0.05. Compared with TAA group, #P < 0.05.

Histological analysis using hematoxylin–eosin (HE) staining revealed that liver lobules in the normal group had a complete structure, clear sinusoids, and no signs of hepatocyte necrosis or inflammatory cell infiltration (Fig. 1A). In contrast, liver samples from the model group exhibited disrupted lobule structures, extensive hepatocyte necrosis, and significant inflammatory infiltration (Fig. 1A). However, treatment with AGK2 mitigated these liver tissue damages in the model group (Fig. 1A). Serum levels of ALT and AST were significantly elevated in the model group compared to the normal group, but these levels were notably decreased in the AGK2 group relative to the model group (Fig. 1B-C).

### AGK2 affected the autophagy and oxidation in ALF mice

Fluorescence microscopy results and western blotting showed that compared with the normal group, the protein level of P-PRKAA1, Beclin1 and LC3B-II were significantly decreased and protein expression of SIRT2 and P62 was significantly increased in model group (Fig. 1D-F). As shown in Fig. 1G-H, compared with the normal group, levels of MDA and TOS were increased in the model group. However, these levels were reduced in the AGK2 group when compared with the model group.

### Effects of different TAA concentrations on cell death rate

**Figure 2:**
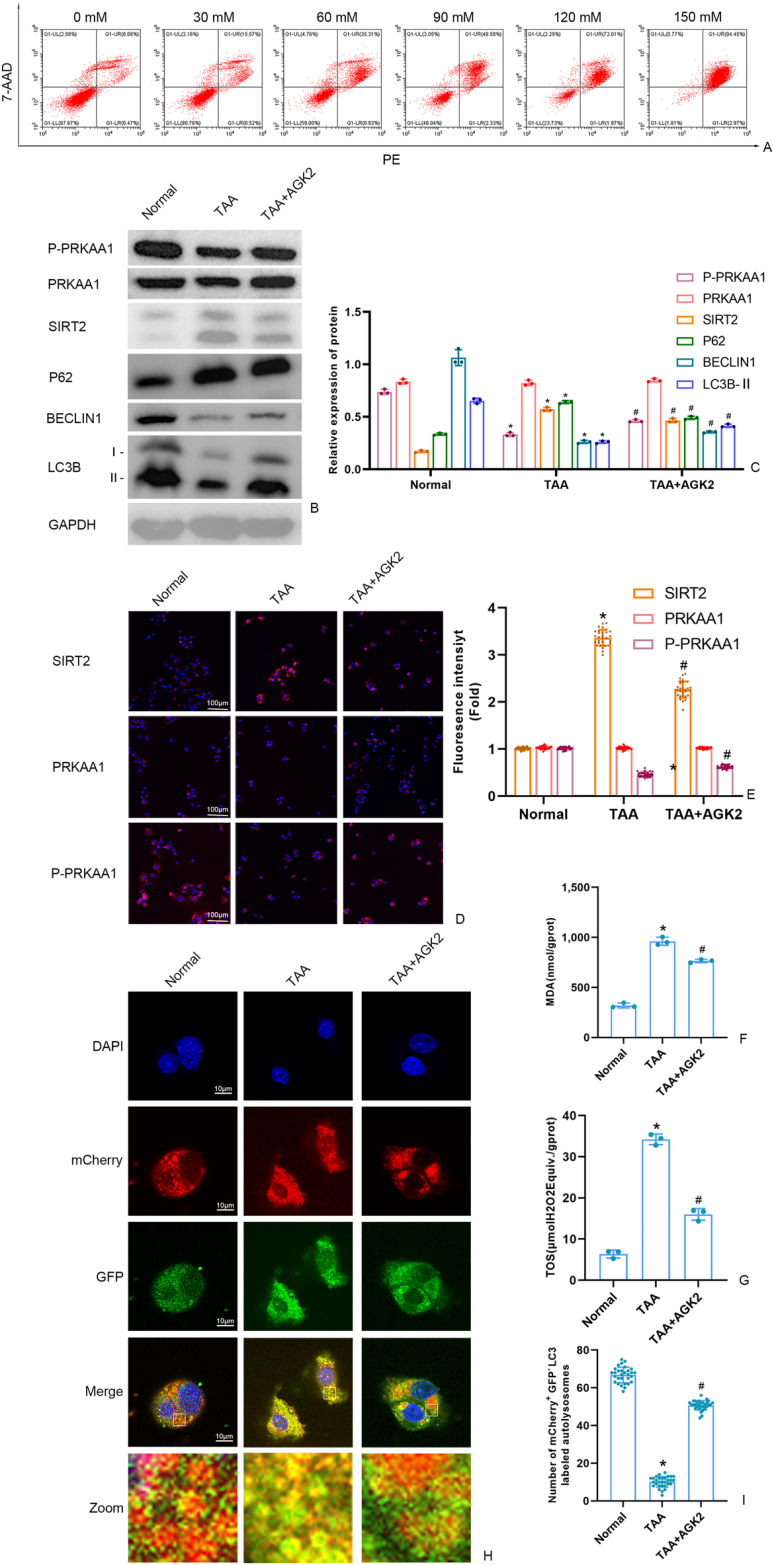
(**A) **The AML12 cell death rate which stimulated by different dosage of TAA was detected by flow cytometry. **(B**,** C**) The SIRT2 protein and autophagy protein level in AML12 cells were measured by Western blot and quantitatively analyzed. Original blots are presented in Supplementary Figure S2. **(D**,** E)** The fluorescence intensity of AML12 cells SIRT2, PRKAA1 and P-PRKAA by immunofluorescence (scale bars = 100 μm). **(F)** Detection of the autolysosomes in AML12 cells by using mCherry-GFP-LC3B observed by confocal microscopy. White boxed regions in the panels are enlarged. (scale bars = 10 μm). **(G)** The mCherry-positive and GFP-negtive LC3-labeled autolysosomes were counted and quantified in at least 30 randomly picked cells. (**H**,** I**) MDA and TOS levels in AML12 cells detected by kits. Data were expressed as the mean ± SD. P values were determined by 2-tailed Student t test. Compared with normal group, *P < 0.05. Compared with TAA group, #*P* < 0.05.

To determine an appropriate treatment dosage of TAA for modeling of AML12 cells, the cell death rate of TAA on AML12 cells were measured by flow cytometry. The rate of cell death in TAA-induced AML12 cells showed a concentration-dependent manner. As shown in Fig. 2A, the rate of PE-positive cells was 9.35%, 16.09%, 36.24%, 50.91%, 73.98% and 97.36% respectively when treated AML12 cell with TAA which dosage of 0 mM, 30 mM, 60 mM, 90 mM, 120 mM, 150 mM. When treated with 90 mM TAA, the cell death rate of AML12 cell had over 50%. Therefore, we selected the 90 mM TAA in following study.

### AGK2 affected the autophagy and oxidation in TAA-induced AML12 cell

Fluorescence microscopy results and western blotting showed that compared with the normal group, the protein level of P-PRKAA1, Beclin1 and LC3B-II were significantly decreased and protein expression of SIRT2 and P62 was significantly increased in model group (Fig. 2B-E). There is no significant difference of the protein level of PRKAA1 among the three groups (Fig. 2B-E). As illustrated in Fig. 2F-G, compared with the normal group, levels of MDA and TOS were increased in the model group. However, these levels were reduced in the AGK2 group when compared with the model group. The mCherry-GFP-LC3B enabled us to measure the autophagy flux in cells. In the case of non-autophagy, mCherry-GFP-LC3B exists in the cytoplasm in the form of diffuse yellow fluorescence; whereas, in the case of autophagy, mCherry-GFP-LC3B aggregates on autophagosome membranes under fluorescence microscopy, and manifests itself in the form of yellow spots. When autophagosomes are fused to lysosomes, they show up as red spots due to partial quenching of GFP fluorescence. Compared with the normal group, The TAA treatment significantly decreased red and yellow point, the decrease of the autophagy flux. Compared with TAA group, the autophagy flux was increased in TAA + AGK2 group (Fig. 2H-I).

### The effect of SIRT2 on P-PRKAA1 and autophagy in TAA stimulated AML12 cell

**Figure 3:**
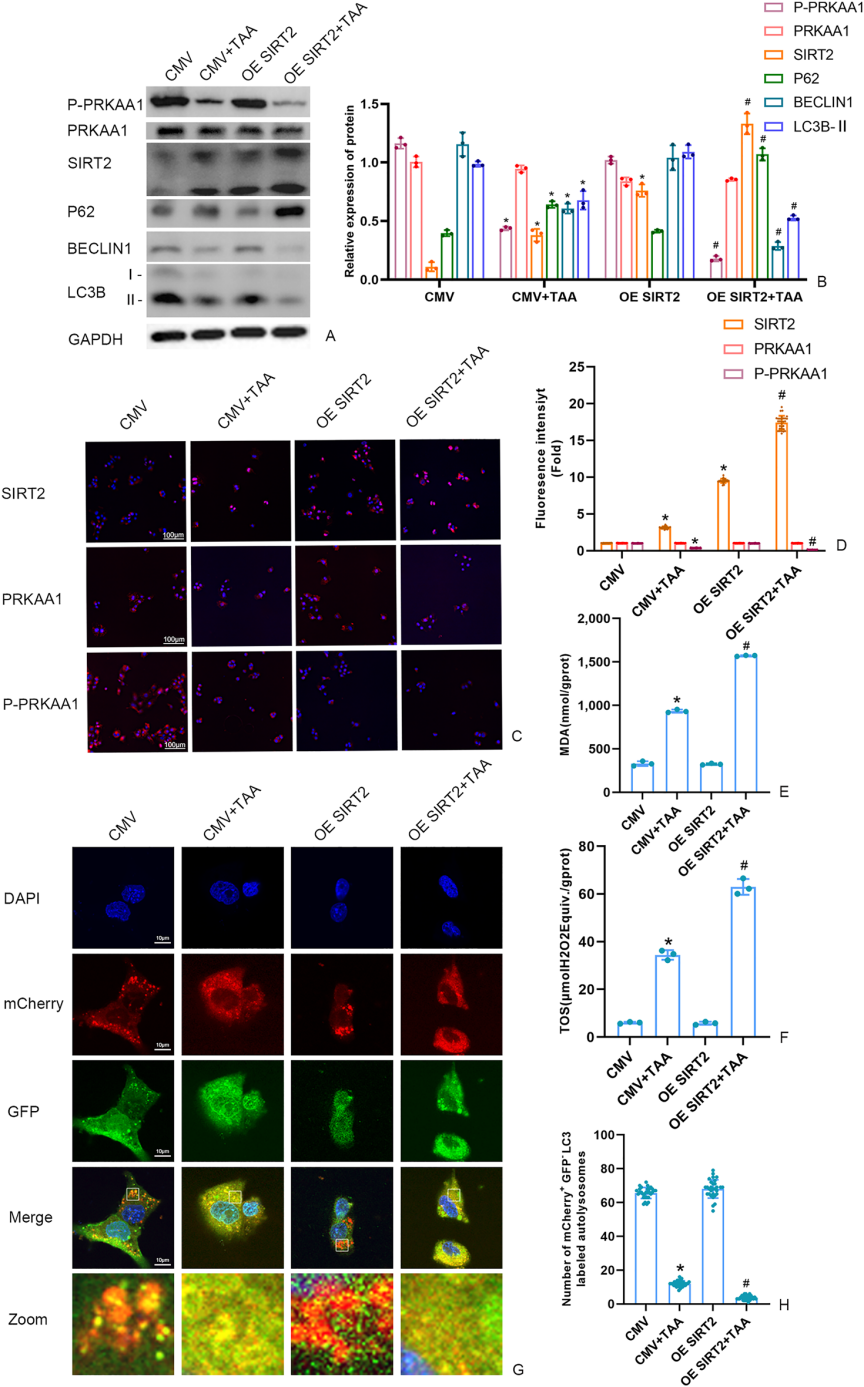
(**A**,** B)** The P-PRKAA1 protein and autophagy protein level in AML12 cells were measured by Western blot and quantitatively analyzed. Original blots are presented in Supplementary Figure S3. **(C**,** D**) The fluorescence intensity of AML12 cells SIRT2, PRKAA1 and P-PRKAA by immunofluorescence (scale bars = 100 μm). **(E)** Detection of the autolysosomes in AML12 cells by using mCherry-GFP-LC3B observed by confocal microscopy. White boxed regions in the panels are enlarged. (scale bars = 10 μm). **(F)** The mCherry-positive and GFP-negtive LC3-labeled autolysosomes were counted and quantified in at least 30 randomly picked cells. (**G**,** H**) MDA and TOS levels in AML12 cells detected by kits. Data were expressed as the mean ± SD. P values were determined by 2-tailed Student t test. Compared with normal group, *P < 0.05. Compared with CMV + TAA group, #P < 0.05.

As depicted in Fig. 3, the OE SIRT2 + TAA group, when compared with the CMV + TAA group, showed a significant decrease in the levels of P-PRKAA1, LC3B-II Beclin1, and the autophagy flux, and an increase in the levels of P62, MDA, and TOS. In Fig. 3A, the Western blot band of SIRT2 was weak, but the immunofluorescence of SIRT2 was strong (Fig. 3C). The strength of the Western blot band intensity are affected by many factors such as exposure time. The trends of the Western blot band as well as the immunofluorescence of SIRT2 was the same in all groups.

**Figure 4:**
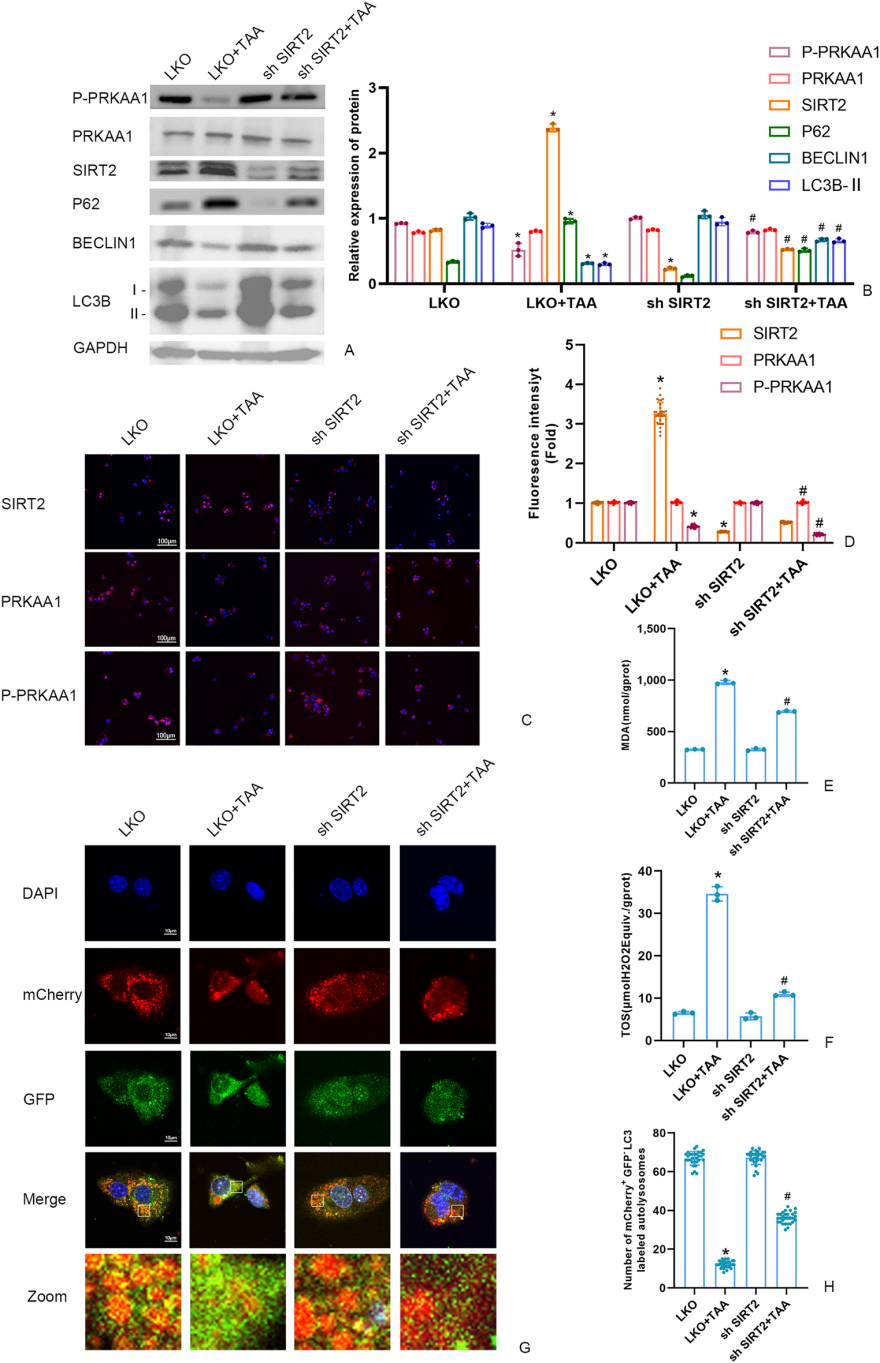
(**A**,** B**) The P-PRKAA1 protein and autophagy protein level in AML12 cells were measured by Western blot and quantitatively analyzed. Original blots are presented in Supplementary Figure S4. **(C**,** D**) The fluorescence intensity of AML12 cells SIRT2, PRKAA1 and P-PRKAA by immunofluorescence (scale bars = 100 μm). **(E)** Detection of the autolysosomes in AML12 cells by using mCherry-GFP-LC3B observed by confocal microscopy. White boxed regions in the panels are enlarged. (scale bars = 10 μm). **(F)** The mCherry-positive and GFP-negtive LC3-labeled autolysosomes were counted and quantified in at least 30 randomly picked cells. (**G**,** H**) MDA and TOS levels in AML12 cells detected by kits. Data were expressed as the mean ± SD. P values were determined by 2-tailed Student t test.Compared with normal group, *P < 0.05. Compared with LKO + TAA group, #P < 0.05.

Conversely, SIRT2 knockdown in the sh SIRT2 + TAA group upregulated levels of P-PRKAA1, LC3B-II, Beclin1, and downregulated P62, MDA, and TOS levels (Fig. 4A-F). Compared to the LKO + TAA group, SIRT2 knockdown significantly increased the autophagy flux (Fig. 4G-H).

### The effect of PRKAA1 on SIRT2 in TAA-induced AML12 cell

**Figure 5:**
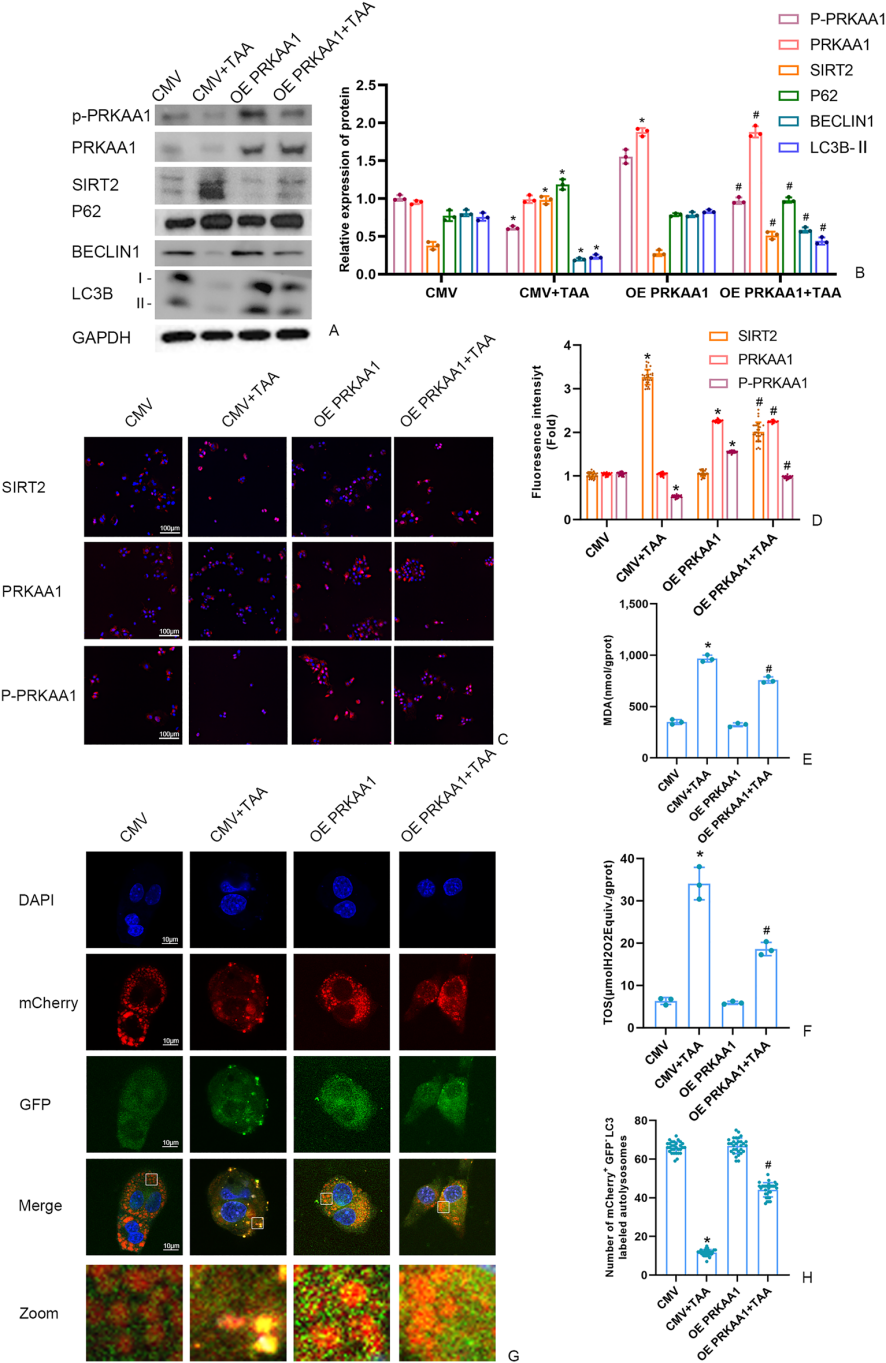
(**A**,** B**) The SIRT2 protein and autophagy protein level in AML12 cells were measured by Western blot and quantitatively analyzed. Original blots are presented in Supplementary Figure S5. **(C**,** D**) The fluorescence intensity of AML12 cells SIRT2, PRKAA1 and P-PRKAA by immunofluorescence (scale bars = 100 μm). **(E)** Detection of the autolysosomes in AML12 cells by using mCherry-GFP-LC3B observed by confocal microscopy. White boxed regions in the panels are enlarged. (scale bars = 10 μm). **(F)** The mCherry-positive and GFP-negtive LC3-labeled autolysosomes were counted and quantified in at least 30 randomly picked cells. (**G**,** H**) MDA and TOS levels in AML12 cells detected by kits. Data were expressed as the mean ± SD. P values were determined by 2-tailed Student t test. Compared with normal group, *P < 0.05. Compared with CMV + TAA group, #P < 0.05.

**Figure 6:**
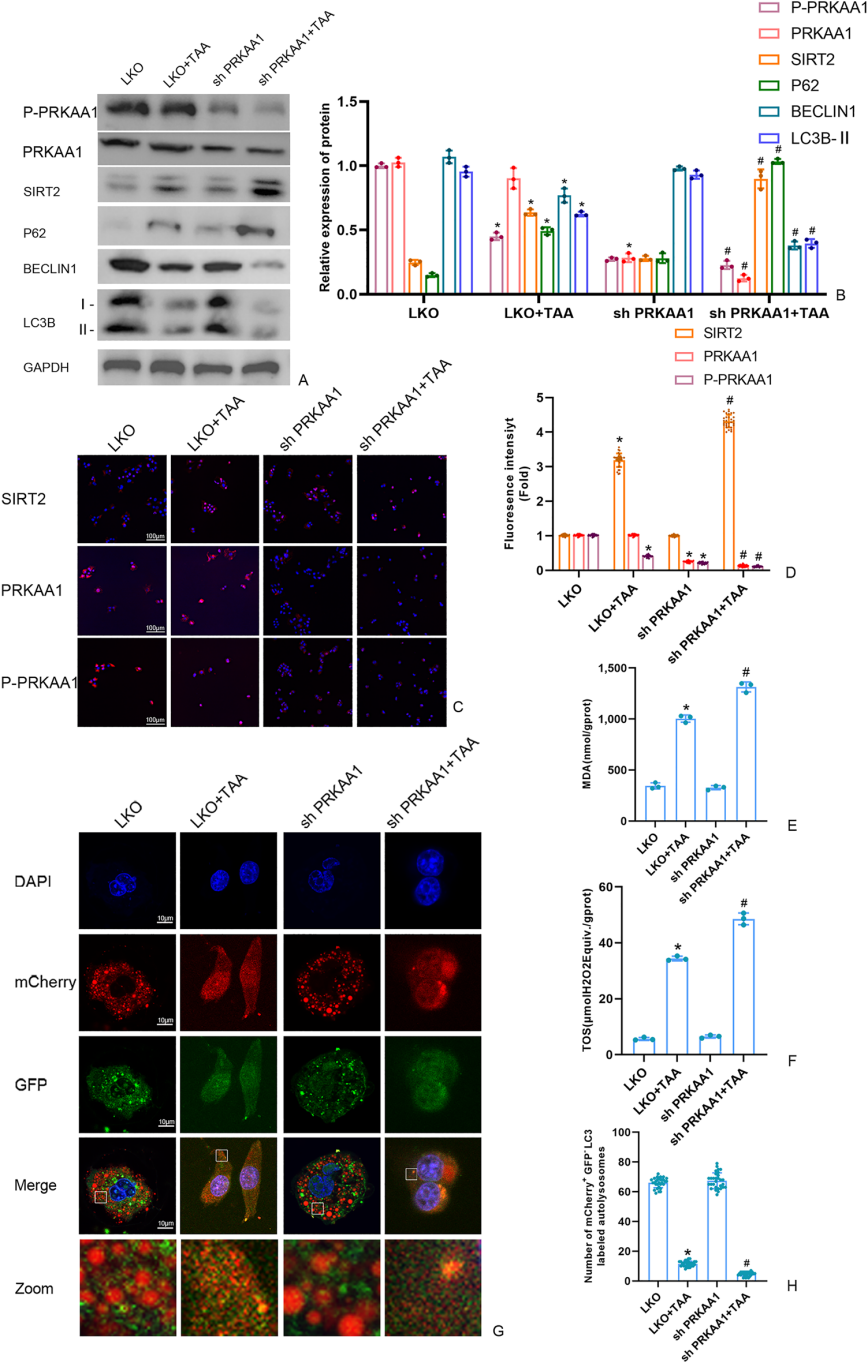
(**A**,** B**) The SIRT2 protein and autophagy protein level in AML12 cells were measured by Western blot and quantitatively analyzed. Original blots are presented in Supplementary Figure S6. **(C**,** D**) The fluorescence intensity of AML12 cells SIRT2, PRKAA1 and P-PRKAA by immunofluorescence (scale bars = 100 μm). **(E)** Detection of the autolysosomes in AML12 cells by using mCherry-GFP-LC3B observed by confocal microscopy. White boxed regions in the panels are enlarged. (scale bars = 10 μm). **(F)** The mCherry-positive and GFP-negtive LC3-labeled autolysosomes were counted and quantified in at least 30 randomly picked cells. (**G**,** H**) MDA and TOS levels in AML12 cells detected by kits. Data were expressed as the mean ± SD. P values were determined by 2-tailed Student t test. Compared with normal group, *P < 0.05. Compared with LKO + TAA group, #P < 0.05.

As shown in Fig. 5, compared with CMV + TAA group, the level of P-PRKAA1, LC3B-II, Beclin1 were significantly promoted in OE PRKAA1 + TAA group. And the autophagy flux was increased in OE PRKAA1 + TAA group. The level of SIRT2, P62, MDA and TOS were reduced in OE PRKAA1 + TAA group. As shown in Fig. 6, PRKAA1 knockdown could up-regulate SIRT2, P62, MDA and TOS level and down-regulate the P-PRKAA1, LC3B-II, Beclin1 level in sh PRKAA1 + TAA group, compared with LKO + TAA group. Compared with LKO + TAA, PRKAA1 knockdown significantly decrease in the autophagy flux (Fig. 6G-H).

## Discussion

Our results indicated that TAA caused significant disruption of liver structure and function in mice and increased the rate of cell death in AML12 cells in a dose-dependent manner. Recent research increasingly supports the vital role of autophagy in the onset and progression of ALF^28,29^. It has been demonstrated that activating autophagy can significantly improve ALF outcomes^30,31^.

Autophagy is a principal cellular degradation process where cytoplasmic materials are transported to and broken down in lysosomes^32^. In our study, TAA could decrease the expression of P-PRKAA1 and the autophagy flux. The overexpression of PRKAA1 decreased P62 and SIRT2 protein levels and enhanced P-PRKAA1, Beclin1, and LC3B-II levels in TAA-induced mice and AML12 cells. Previous studies have reported that enhancement of AMPK/glycogen synthase kinase-3β (GSK3β)/Nuclear factor-erythroid 2-related factor 2 (Nrf2) signalling pathway protects against APAP-induced ALF^33,34^.

Sirtuin 2 (SIRT2), a member of the sirtuin family, is characterized by its evolutionarily conserved NAD + -dependent deacetylase activity^35^. The SIRT2 is predominantly found in metabolically active tissues such as the liver, heart, skeletal muscle, and brain^14^. It binds to and deacetylates FoxO3a under oxidative stress conditions, potentially enhancing the expression of FoxO (Forkhead-box class O) target genes such as p27, manganese superoxide dismutase, and Bim^36^. FoxO transcription factors are known to activate mTORC1 (mechanistic target of rapamycin complex 1) by inducing sestrin 3 (Sesn3), which promotes AMPK and inhibits mTORC1, thus relieving the blockade on autophagy initiation^37^. Previous studies have shown that deletion of SIRT2 reduces the AMPK response to metformin in mice treated with Ang II in the mice model of pathological cardiac hypertrophy^18^. In acute ethanol-exposed macrophages, knockdown of SIRT2 led to increasing LC3 activation^38^. SIRT2 inhibitor NCO-90/14 increases autophagosome accumulation and LC3-II levels in cell lines^39^. In our study, the knockdown of SIRT2 decreased P62 protein levels and enhanced P-PRKAA1, Beclin1, and LC3B-II levels in TAA-induced AML12 cells.

**Figure 7:**
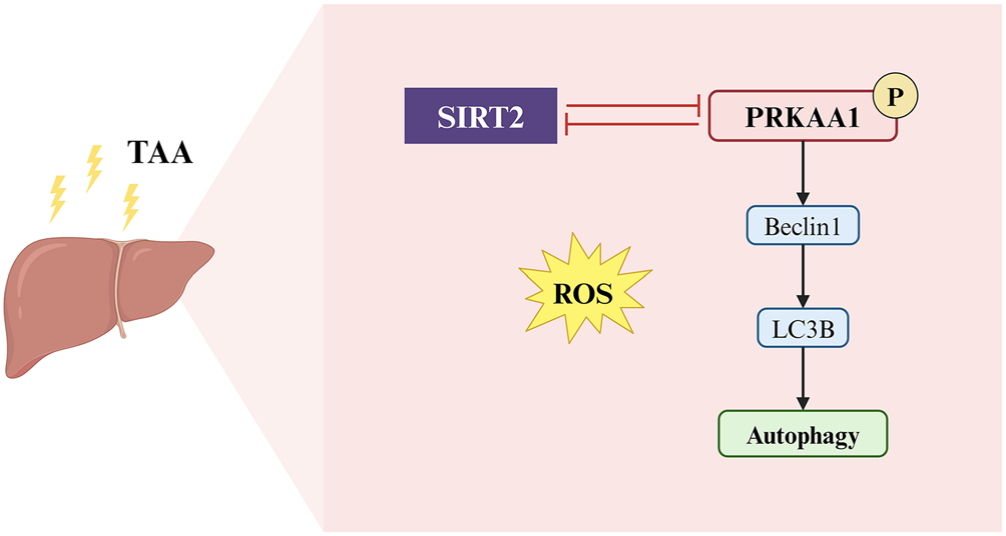
Summary diagram. The SIRT2-AMPK axis regulates autophagy in acute liver failure. TAA: thioacetamide; SIRT2: Sirtuin 2; PRKAA1: protein kinase AMP-activated alpha 1 catalytic subunit; LC3B: Microtubule-Associated Protein 1 Light Chain 3B; ROS: reactive oxygen species.

Using both in vivo and in vitro models of ALF induced by TAA, our study explored the effects of SIRT2 inhibition AGK2 and the modulation of PRKAA1 expression through overexpression and knockdown approaches. Histopathological analyses revealed that AGK2 ameliorated liver damage in ALF mice, evidenced by reduced hepatocyte necrosis and inflammatory infiltration. Kits showed that AGK2 treatment lowered serum ALT and AST levels, indicating reduced hepatic injury. Knockdown of SIRT2 in ALF models led to decreased P62 protein levels, while enhancing P-PRKAA1, Beclin1, and LC3B-II levels, indicating a protective role against autophagy and oxidative stress. Similarly, overexpression of PRKAA1 resulted in decreased SIRT2 levels and reduced markers of oxidative stress, suggesting a regulatory effect of PRKAA1 on SIRT2 and autophagy related pathways. The possible mechanisms were showed as Fig. 7.

## Conclusion

Based on above results, our data have verified that SIRT2 plays a significant role in the progression of acute liver failure, influencing key pathways related to autophagy and autophagy. AGK2, as a SIRT2 inhibitor, demonstrated hepatoprotective effects in ALF models by modulating the expression of autophagy-related proteins and reducing oxidative stress markers. Furthermore, our study highlighted the reciprocal regulatory relationship between SIRT2 and PRKAA1/AMPKα, underscoring the complexity of molecular interactions in ALF. The findings suggest that targeting the SIRT2-AMPK axis could be a potential therapeutic strategy in ALF management, offering insights into the molecular mechanisms underlying liver injury and repair. Our study opens up new possibilities for future research exploring targeted interventions for ALF and related liver diseases.

### Supplementary Information

Supplementary Information.

## Data Availability

Data is provided within the manuscript or supplementary information files.
