# Supplementary material for: The SIRT2-AMPK axis regulates autophagy induced by acute liver failure
Source: Sci Rep. 2024 Jul 15;14:16278. doi: 10.1038/s41598-024-67102-w (PMC11251177; doi:10.1038/s41598-024-67102-w)
Supplement: Supplementary file 1 — Supplementary Information. [file 41598_2024_67102_MOESM1_ESM.pdf]

The SIRT2-AMPK axis regulates autophagy induced by acute liver failure

Qingqi Zhang, Jin Guo, Chunxia Shi, Danmei Zhang, Yukun Wang, Luwen Wang, Zuojiong Gong

Figure 1C

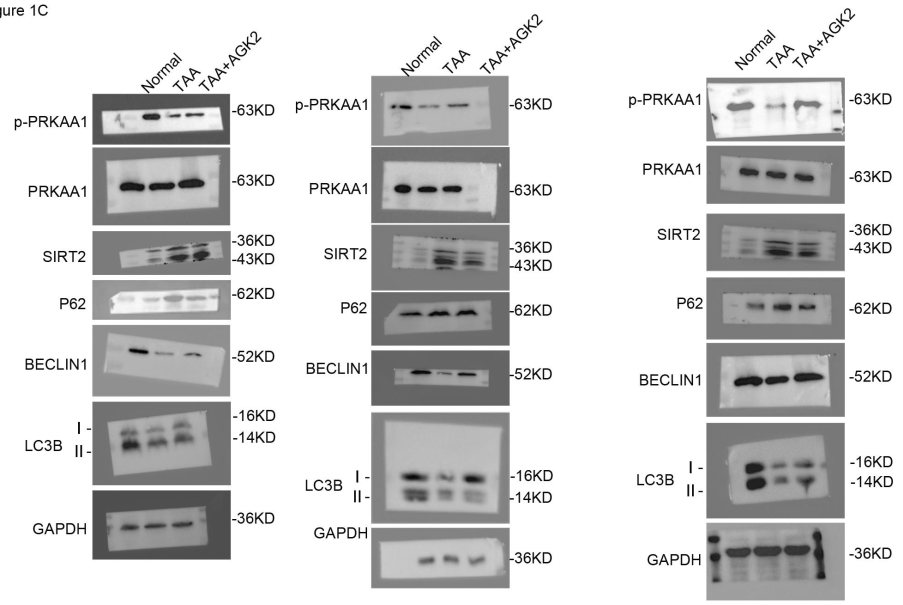

Fig.S1. Original image of the blot in Figure 1C.

Figure 2B

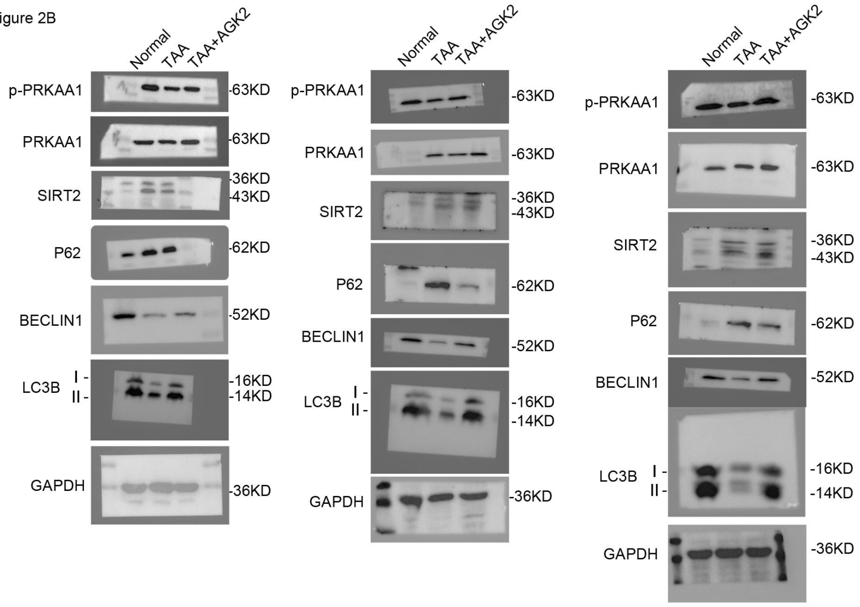

Fig.S2. Original image of the blot in Figure 2B.

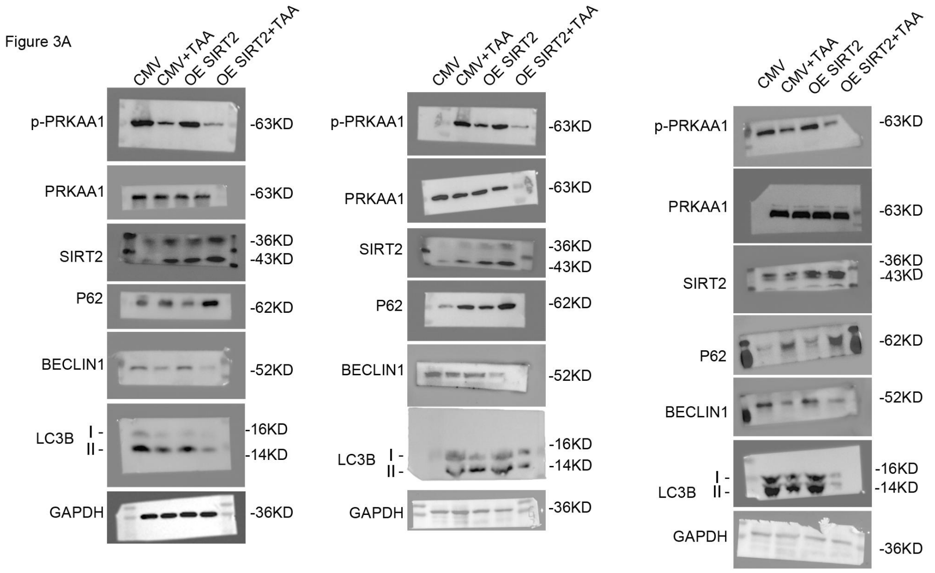

Fig.S3. Original image of the blot in Figure 3A.

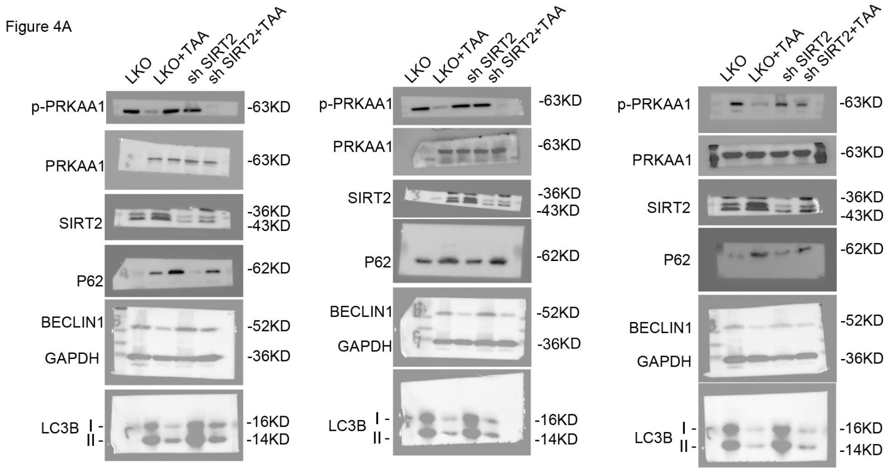

Fig.S4. Original image of the blot in Figure 4A.

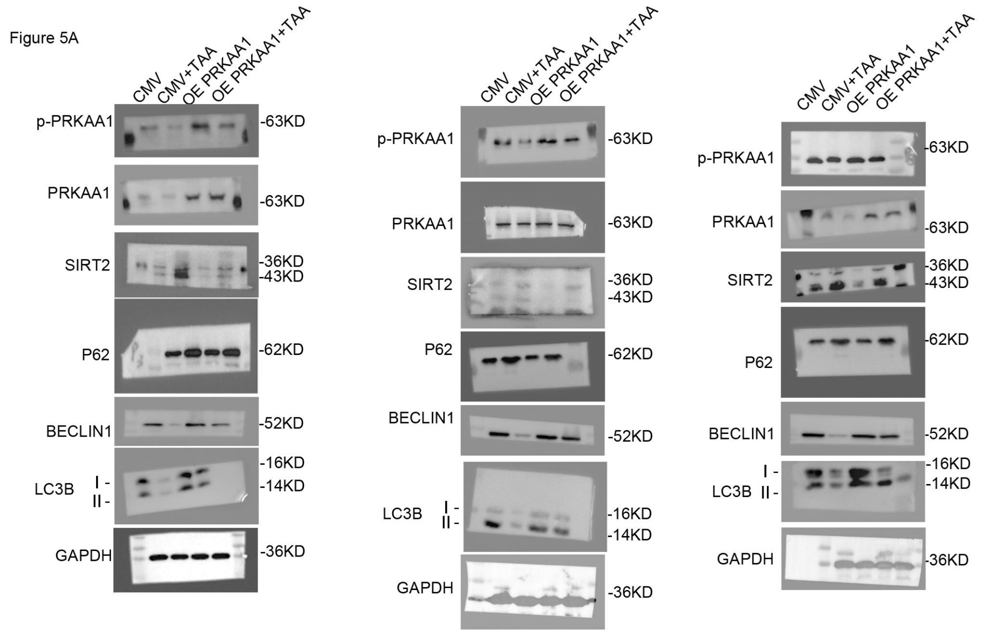

Fig.S5. Original image of the blot in Figure 5A.

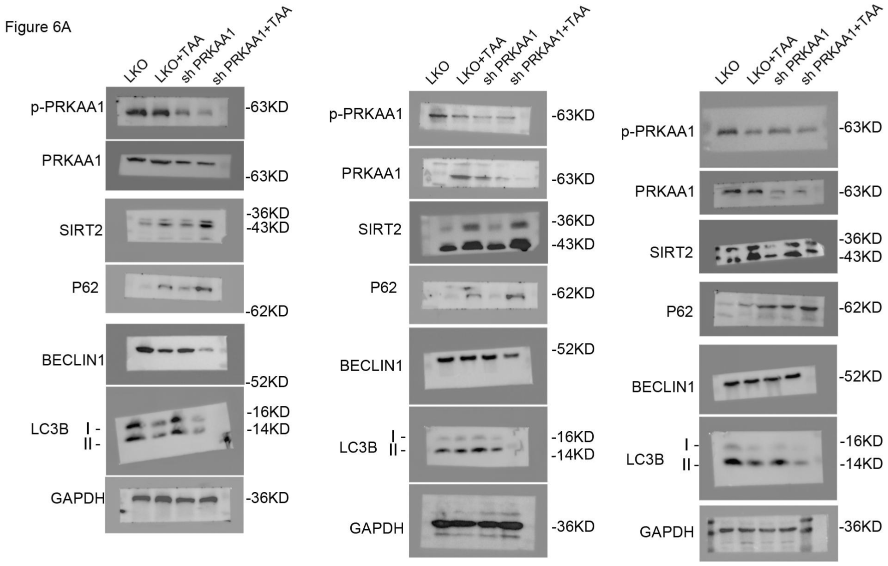

Fig.S6. Original image of the blot in Figure 6A.
